# The impact of *Tetracapsuloides bryosalmonae* and *Myxobolus cerebralis* co-infections on pathology in rainbow trout

**DOI:** 10.1186/s13071-017-2347-6

**Published:** 2017-09-25

**Authors:** Mohamed H. Kotob, Bartolomeo Gorgoglione, Gokhlesh Kumar, Mahmoud Abdelzaher, Mona Saleh, Mansour El-Matbouli

**Affiliations:** 1 Clinical Division of Fish Medicine, University of Veterinary Medicine, Veterinärplatz 1, 1210 Vienna, Austria; 2 Department of Pathology, Faculty of Veterinary Medicine, Assiut University, Assiut, 71526 Egypt; 3 Department of Biological Sciences, University of Toledo, Toledo, OH 43606 USA

**Keywords:** Proliferative kidney disease, Whirling disease, Co-infection, *Oncorhynchus mykiss*, Histopathology

## Abstract

**Background:**

Myxozoan parasites pose emerging health issues for wild and farmed salmonid fish. Rainbow trout (*Oncorhynchus mykiss*) is a particularly susceptible species to *Tetracapsuloides bryosalmonae* (Malacosporea), the etiological agent of Proliferative Kidney Disease (PKD), and to *Myxobolus cerebralis* (Myxosporea), the etiological agent of Whirling Disease (WD). The objective of this study was to investigate the impact of myxozoan co-infections on the pathogenesis of PKD and WD in the rainbow trout.

**Methods:**

Two groups of rainbow trout (96 fish each) were primarily infected with *T. bryosalmonae* and triactinomyxons of *M. cerebralis*; after 30 days half of the fish in each group were co-infected with these parasites vice versa and remaining half was continued as single infection. Mortalities and clinical signs were recorded at different time points. Histopathology and immunohistochemistry were performed to assess the extent of each infection and estimate the parasite burden between groups.

**Results:**

Fish firstly infected with *M. cerebralis* and co-infected with *T. bryosalmonae* exhibited exacerbated pathological changes of both parasitic diseases and elicited a higher mortality rate. A higher kidney swelling index (grade 4) appeared together with more severe cartilage destruction and displacement, when compared to the pathological changes in fish upon single infections with *T. bryosalmonae* or *M. cerebralis*. Conversely, fish firstly infected with *T. bryosalmonae* and co-infected with *M. cerebralis* also exhibited typical pathological changes of both parasitic diseases, but with a lower mortality rate, similar as caused by the single *T. bryosalmonae* or *M. cerebralis* infection. WD clinical signs were milder, without skeletal deformities, while kidney swelling index was similar to single infection with *T. bryosalmonae* (grade 2 to 3).

**Conclusions:**

In this study, a co-infection with myxozoan parasites was for the first time successfully achieved in the laboratory under controlled conditions. The impact of co-infections in concurrent myxozoan infections mainly depends on the primary pathogen infecting the host, which could alter the outcomes of the secondary pathogen infection. The primary *M. cerebralis* infection followed by *T. bryosalmonae* had a much more serious impact and elicited a synergistic interaction. Contrasting results were instead seen in rainbow trout primarily infected with *T. bryosalmonae* and then co-infected with *M. cerebralis*.

## Background

Proliferative Kidney Disease (PKD) and Whirling Disease (WD) are the main issues posed by myxozoan parasites to wild and farmed salmonid fish. PKD is caused by the malacosporean parasite *Tetracapsuloides bryosalmonae* [1, 2] and currently considered as an emerging disease in Europe and North America [3]. In farmed rainbow trout (*Oncorhynchus mykiss*) mortality can range from 20%, in uncomplicated cases, up to 95–100% in outbreaks complicated by secondary infections and stressing conditions [4–7] or when occurring at higher water temperatures [8–10].

PKD was responsible for a long term decrease in brown trout (*Salmo trutta*) populations in many European countries especially Switzerland [11–14]. Recently, it was linked to the multifactorial etiology of Black Trout Syndrome, decimating autochthonous brown trout in Austria [8]. The life-cycle of *T. bryosalmonae* includes freshwater bryozoans, *Fredericella sultana* (Phylactolaemata), as the definitive host and salmonids as the intermediate host [15, 16]. Kidney and spleen are the main target organs, in which the celozoic sporogonic proliferation (in the lumen of renal tubules and mesonephric ducts) elicits the formation of mature malacospores; but the histozoic extrasporogonic proliferation (in the interstitium) causes a tumor-like chronic lymphoid hyperplasia [6, 17–21].

WD is a highly debilitating parasitic disease of salmonid fish, caused by the myxosporean parasite *Myxobolus cerebralis* [22]. The two-host life-cycle of *M. cerebralis* includes salmonid fish and an oligochaete worm, *Tubifex tubifex*, releasing triactinomyxon spores infective for salmonids [23]. *Myxobolus cerebralis* spores proliferate and sporulate within the fish cartilage, as a target site causing severe dysplasia followed by granulomatous reactions and cartilage destruction; thus, WD is characterized by pathognomonic skeletal deformities [24].

Co-infections have serious effects on the host such as changing the host susceptibility to different pathogens, infection duration and altering their pathogenic course [25, 26]. The interaction occurring between different pathogens during co-infection could be either synergistic, i.e. the presence of one pathogen may enhance subsequent infections and increasing the host susceptibility to other pathogens or antagonistic, i.e. the presence of one pathogen may inhibit and compete with subsequent infections by other pathogens affecting their occurrence and pathogenesis [25–27]. A case study of co-infection by *Nucleospora cyclopteri* (Microsporidia) and *Kudoa islandica* (Myxozoa) in farmed lumpfish, *Cyclopterus lumpus* L., was reported in Norway, with mortality rates up to 65% [28]. Co-infections with sympatric myxozoans frequently occur in salmonids, showing a variable degree of host competition; including mixed infection of myxozoan species (*T. bryosalmonae*, *Sphaerospora truttae*, *Chloromyxum schurovi, C. truttae* and *Myxobolus* sp.) which was detected in farmed brown trout in Scotland [29]. Moreover, the initial *T. bryosalmonae* infection strongly influences the sub-sequential development of *C. schurovi* [29, 30].

The fact that PKD and WD will increase their incidence in wild and farmed salmonids due to environmental changes concerns the aquaculture and fishery representatives. PKD is currently considered an emerging disease. New outbreaks were recently described in new geographical areas such as in central Europe in Switzerland [12], Norway [31], Estonia [32], Slovenian rivers [33] and Austria [8]. More recently, a PKD outbreak has been reported in August 2016 in Montana’s Yellowstone River in the USA [34], where WD has also been reported to decrease rainbow trout population since the beginning of the twenty-first Century [35–37].

The aim of this study was to investigate the impact of co-infections with the myxozoan parasites *T. bryosalmonae* and *M. cerebralis* on rainbow trout. The differential host susceptibility was investigated in terms of mortality rate, clinical signs and pathological lesions.

## Methods

### Fish maintenance

Specific pathogen free rainbow trout approximately 2 months post-hatching (mean length 4.02 ± 0.26 cm, mean weight 0.6 ± 0.15 g) were obtained from a disease-free certified hatchery (Trout Farm GLÜCK). Fish were acclimatized for 1 week in a 500 l glass tank in a flow-through system supplied with dechlorinated tap water (approx. 1 l/m; pH 7.0–7.2; dissolved oxygen 9.0–10.0 mg/l; nitrite 0 mg/l; nitrate 8–10 mg/l; total hardness 179 mg/l) at 16 ± 1 °C with adequate aeration provided. The fish room has artificial - as well as day light (12 h light /12 h dark). Feeding rate was adjusted to 1% BW/day using a commercial pellet (Garant Aqua, Pöchlarn, Austria). Before starting the experiment, 10 fish were randomly sampled during the tank allocation and examined for the presence of any ecto- or endoparasites using light microscopy examinations of mucus smears from skin, gills and gut. Bacterial examinations were also carried out using routine bacteriology from kidney and spleen. The specific presence of *T. bryosalmonae* or *M. cerebralis* was tested in the internal organs, including from cranium by using conventional PCR using PKD primers, *T.b*.18S_F (5′-GGA CAC TGC ATG TGC TGC ATA GT-3′) and *T.b.*18S_R (5′-CCA TGC TAG AAT GTC CAG GCA CT-3′) [21] and *M. cerebralis* primers, Tr5–17 (5′-GCC CTA TTA ACT AGT TGG TAG TAT AGA AGC-3′), Tr3–17 (5′-GGC ACA CTA CTC CAA CAC TGA ATT TG-3′) [38]. All the tested fish were found to be free from ectoparasites, endoparasites and bacteria.

### *Tetracapsuloides bryosalmonae* spores

*Tetracapsuloides bryosalmonae* spores were collected from our laboratory infected *F. sultana* colonies [20, 39]. Briefly, infected colonies were dissected, mature parasite spore sacs were collected, and a spore suspension was prepared in dechlorinated tap water according to Kumar et al. [20].

### Triactinomyxon spores of *Myxobolus cerebralis*

Triactinomyxon (TAM) spores were collected through a 20 μm mesh sieve from our laboratory infected *T. tubifex* that had been exposed previously to spores of *M. cerebralis* obtained from infected rainbow trout [24, 40].

### Experimental infection of fish

**Fig. 1:**
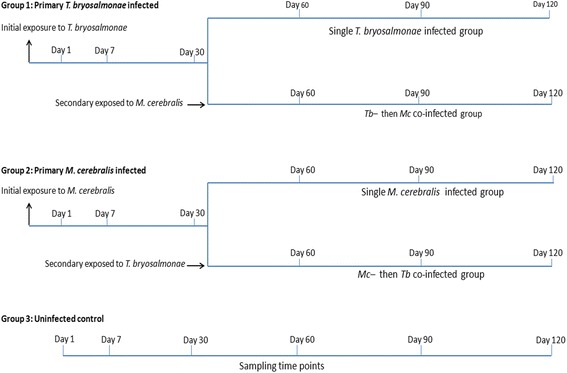
Schematic experimental design. This design shows three groups: primary *T. bryosalmonae*-infected, *M. cerebralis*-infected and uninfected control and different sampling time points

The full experimental design is summarized in Fig. 1. After acclimation, rainbow trout were allocated in three groups.

Group 1: 96 rainbow trout were exposed to free *T. bryosalmonae* spores released from 22 mature parasite sacs at 16 °C according to Kumar et al. [20]. Eight fish were sampled at 1, 7 and 30 days post-exposure (dpe). After 30 days, the remaining 72 fish were divided into two groups (36 fish each) and allocated into separated 60 l aquaria. One group continued as single *T. bryosalmonae-*infected group; the other one was infected with triactinomyxon spores of *M. cerebralis* (2000 TAMs per fish) for 2 h at 16 °C as co-infection and continued as *T. bryosalmonae*-then *M. cerebralis* co-infected group (*Tb*-then *Mc* co-infected group). Eight fish were sampled from each group at 60, 90 and 120 dpe.

Group 2: Similar to group 1, 96 rainbow trout were exposed to triactinomyxons (2000 TAMs/fish). After 30 min, two fish were sampled to be examined histologically for the presence of penetrated stages of the triactinomyxon spores of *M. cerebralis* through the epidermis. Eight fish were sampled at 1, 7 and 30 dpe. At 30 days, the remaining fish were equally divided into two groups and allocated into separated 60 l aquaria. One group continued as single *M*. *cerebralis-*infected group; the other one was infected with free *T. bryosalmonae* spores released from 11 mature parasite sacs as co-infection and continued as *Mc*-then *Tb* co-infected group. Eight fish were sampled from each group at 60, 90 and 120 dpe.

Group 3: 60 rainbow trout were allocated in two 60 l flow-through aquaria (30 fish/tank) and kept as an uninfected control group. Eight fish were sampled at 1, 7, 30, 60, 90 and 120 dpe.

### Fish sampling

Following experimental exposures, fish were maintained in all tanks under the same conditions such as water parameters, temperature and photoperiod applied as described earlier and monitored three times daily for the appearance of any clinical signs and mortalities were recorded. Fish were sampled at 1, 7, 30, 60, 90, 120 dpe and euthanized using an overdose of tricaine methanesulphonate (500 mg/l, MS-222, Sigma-Aldrich, Steinheim, Germany). However, fish showed signs of morbidity and fish that showed skin darkening, lethargy were immediately euthanatized (Endpoint in our animal study proposal).

Total body length (TBL) and total body weight (TBW) were recorded. Samples from posterior kidney, spleen, liver, gills, cranium, vertebral column and brain were divided into 2 portions; one fixed in 10% neutral buffered formalin for 24 h and the other preserved in RNA later (Sigma-Aldrich). The kidney swelling index was used to assess the extent of kidney enlargement during PKD, according to Clifton-Hadley et al. [17].

### Histology and immunohistochemistry

Tissue samples were processed for histology, 5 μm sections were stained with hematoxylin and eosin (H&E) or processed for immunohistochemistry (IHC). The severity of PKD was graded and assessed in H&E stained sections using the PKD histological assessment, with a grading scale from 1 to 3 according to Peeler et al. [30]. WD lesions were evaluated according to grading scale described by Baldwin et al. [41]. Immunohistochemistry for different tissues at all time points was carried out to assess the presence and burden of *T. bryosalmonae* in the kidneys in single *T. bryosalmonae* infected and in co-infected groups. Tissue sections were incubated with *T. bryosalmonae* monoclonal antibody P01 (1:10 diluted) (Aquatic Diagnostics, Stirling, UK), following the procedures previously described [20]. The antibody-antigen reaction was visualized using a Dako EnVision + System-HRP (AEC) kit (Dako, Carpinteria, USA). Sections were counterstained with hematoxylin and examined for the presence of any parasite stage using an Olympus BX53 microscope. The number of visualized parasites was counted in 10 microscopic fields (at 400× magnifications) in 3 posterior kidney compartments, including the interstitium, blood vessels and lumen of the kidney tubules, as described by Schmidt-Posthaus et al. [42].

### Statistical analysis

Data obtained from the measurement of total body lengths, total body weights and *T. bryosalmonae* parasite numbers were statistically analyzed to detect significant differences between groups (mean ± SD) by one way ANOVA and LSD using IBM SPSS software version 24 (IBM Corporation). Significant differences between percentages of mortality in single *T. bryosalmonae*, single *M. cerebralis* and co-infected groups were analyzed using two-sample *t*-test (Statistics calculator software). Differences were considered significant when *P* < 0.05.

## Results

### Clinical signs, gross lesions and mortalities

**Fig. 2:**
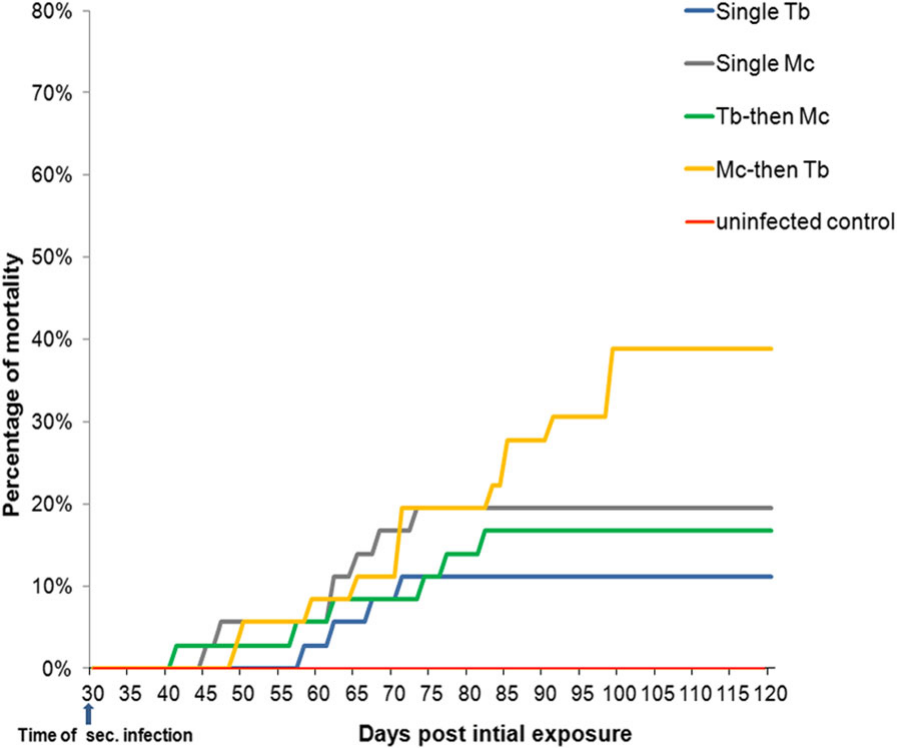
Mortality percentages of rainbow trout. Mortality was recorded in the all groups after the co-infection onset

During the initial 30 days after primary parasite infections, 2 fish died from single *Tb* infected group and 5 from single *Mc* infected group. After secondary infections the highest mortalities were observed in *Mc-*then *Tb* co-infected group when compared to all infected groups. Data of mortality rates are shown in (Fig. 2).

Fish TBL showed no significant difference between single- and co-infected groups, when compared to the uninfected control group at 1 dpe (*F*_(2,21)_ = 0.435, *P* = 0.653), 7 dpe (*F*_(2,21)_ = 0.618, *P* = 0.549), 30 dpe (*F*_(2,21)_ = 1.181, *P* = 0.327) and 60 dpe (*F*_(4,35)_ = 0.677, *P* = 0.612). The TBW showed also no significant difference between single- and co-infected groups, when compared to the uninfected control group at 1 dpe (*F*_(2,21)_ = 0.317, *P* = 0.732), 7 dpe (*F*_(2,21)_ = 0.203, *P* = 0.818), 30 dpe (*F*_(2,21)_ = 1.763, *P* = 0.196) and 60 dpe (*F*_(4,35)_ = 1.417, *P* = 0.249). The TBL of *Mc*-then *Tb* co-infected group was significantly affected at 90 dpe (*F*_(4,35)_ = 4.717, *P* < 0.01) and 120 dpe (*F*_(4,35)_ = 10.181, *P* < 0.001). Moreover, the TBW of this group was also significantly affected at 90 dpe (*F*_(4,35)_ = 38.658, *P* < 0.001) and 120 dpe (*F*_(4,35)_ = 77.736, *P* < 0.001) when compared to single *Tb*, single *Mc*, *Tb*-then *Mc* co-infected and uninfected control groups.

**Fig. 3:**
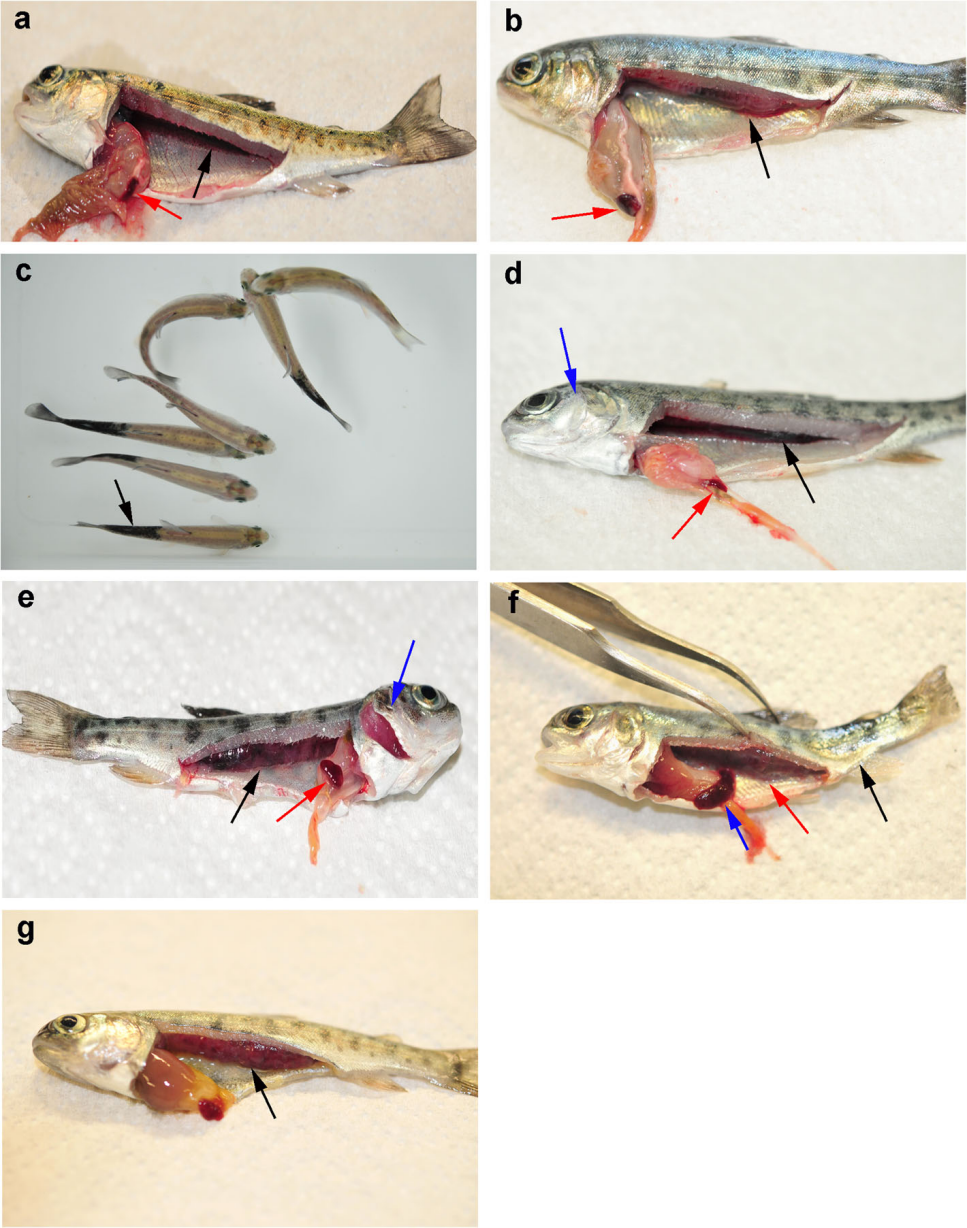
Comparative clinical appearance of PKD and WD on rainbow trout during single and co-infection. **a** Uninfected control fish showing normal kidney (*black arrow*) and spleen (*red arrow*). **b** Single *Tb* infected fish showing enlargement in the kidney (*black arrow*) and spleen (*red arrow*) at 90 dpe. **c** Single *Mc* infected group at 60 dpe showing some fish exhibited black tail (*arrow*). **d** Single *Mc* infected group at 90 dpe showing deformed gill operculum (*blue arrow*), normal kidney (*black arrow*) and normal spleen (*red arrow*). **e** *Mc*-then *Tb* co-infected fish showing swollen kidney (*black arrow*) and spleen (*red arrow*) together with shortening and deformities of gill operculum (*blue arrow*) at 90 dpe to *Mc* (60 dpe to *Tb*). **f** *Mc*-then *Tb* co-infected fish showing bowing of caudal area and skeletal deformities (*black arrow*) and severely enlarged kidney (*red arrow*) and spleen (*blue arrow*), at 120 dpe to *Mc* (90 dpe to *Tb*). **g** *Tb*-then *Mc* co-infected fish showing severe enlarged kidney (*black arrow*), pale liver but no signs of whirling disease were noticed, 120 dpe to *Tb* (90 dpe to *Mc*)

Internal PKD lesions were observed after 60 dpe with mild renal hypertrophy and splenomegaly. At 90 dpe, the kidneys swelling became more obvious in the single *Tb* infected group (Fig. 3b). There was no remarkable difference in terms of either gross lesions or kidney swelling index (grade 2) between single *Tb* infected and *Tb*-then *Mc* co-infected groups at 60 and 90 dpe. However, in *Mc*-then *Tb* co-infected group, kidneys and spleens were more severely enlarged, up to grade 3 (Fig. 3e).

At 120 dpe, kidneys and spleens were severely enlarged in all *T. bryosalmonae* infected groups whereas this enlargement was more and massive in *Mc*-then *Tb* co-infected group, showing a granulomatous-like appearance, scored as grade 4 (Fig. 3f) and ascites was also noticed in moribund fish from this group.

Clinical signs of WD were observed after 40 dpe in 30% of single *Mc* infected group and in 60% of *Mc*-then *Tb* co-infected group. These signs were in the form of whirling movements with an appearance of slight shortening of gill operculum. At 60 dpe, fish showed black tails (Fig. 3c) and the percentage reached up to 60% in *Mc*-then *Tb* co-infected group, when compared to 44% in single *Mc* infected group.

At 90 and 120 dpe, *Mc*-then *Tb* co-infected group showed 100% whirling movements and 90% deformities in the cranium and caudal area (Fig. 3e, f). Whereas single *Mc* infected group showed 70% whirling movements and 35% cranial deformities (Fig. 3d). On the other hand, fish from *Tb*-then *Mc* co-infected group showed milder clinical signs of whirling disease. In this group 18% of fish exhibited whirling movements which appeared after 50 dpe from secondary infection with *M. cerebralis.* Until the end of the experiment (120 dpe), fish of this group did not show any signs of cranial deformities, caudal curvature or black tail (Fig. 3g).

### Histopathological assessment

Tissue samples collected from single *Tb* and *Mc* infected groups at 1 and 7 dpe did not show any evidence of pathological changes. Extrasporogonic stages of *T. bryosalmonae* were first detected in the kidneys at 30 dpe. Data of renal lesion scores and *T. bryosalmonae* numbers are summarized in Table 1.

**Table 1:** Comparison of *T. bryosalmonae* numbers in immunohistochemical stained tissues and histopathological lesion scores in different groups. Presented data are the mean values of *T. bryosalmonae* numbers from immunohistochemical stained tissues and the results of histopathological lesion scores from kidneys samples at 60, 90 and 120 dpe

	60 dpe to *T. bryosalmonae*			90 dpe to *T. bryosalmonae*			120 dpe to *T. bryosalmonae*	
	Single *Tb* infected group	*Tb*-then *Mc* co-infected group	*Mc*-then *Tb* co-infected group	Single *Tb* infected group	*Tb*-then *Mc* co-infected group	*Mc*-then *Tb* co-infected group	Single *Tb* infected group	*Tb*-then *Mc* co-infected group
*T. bryosalmonae* numbers by IHC								
1-Interstitium	43.71 ± 21.17 (20–74)^b^	39.5 ± 21.67 (24–87)^b^	99.4 ± 43.24 (45–164)^a^	58.71 ± 30.17 (18–94)^b^	68.6 ± 12.09 (55–82)^b^	156.0 ± 36.03 (98–192)^a^	90.83 ± 21.79 (68–120)^a^	121.67 ± 14.57 (110–138)^a^
2-Blood vessels	0.71 ± 1.25 (0–3)^a^	5.00 ± 10.62 (0–31)^a^	5.2 ± 4.15 (0–11)^a^	2.86 ± 1.86 (0–5)^b^	2.8 ± 1.92 (0–5)^b^	8.0 ± 4.0 (2–12)^a^	3.83 ± 1.83 (2–7)^a^	3.33 ± 1.53 (2–5)^a^
3-Interepithelial	0.43 ± 0.79 (0–2)^a^	0.5 ± 1.07 (0–3)^a^	0.6 ± 0.89 (0–2)^a^	0.0 (0–0)^b^	0.0 (0–0)^b^	2.2 ± 1.3 (0–3)^a^	0.0 (0–0)^a^	0.0 (0–0)^a^
Histopathological lesions								
A-Interstitium								
1-Infiltration of inflammatory cells	3 (2–4)	3 (2–5)	4 (3–5)	4 (4–5)	5 (4–5)	6 (4–6)	5 (5–6)	6 (5–6)
2-Fibosis	3 (2–5)	3 (2–5)	4 (3–5)	4 (4–5)	4 (3–5)	6 (4–6)	5 (4–6)	6 (5–6)
3-Haemorrhage	1 (1–2)	3 (2–4)	4 (3–5)	3 (3–4)	4 (4–5)	3 (2–3)	2 (2–3)	2 (2–3)
4-Parasites with daughter cells	3 (2–5)	3 (2–4)	4 (4–5)	4 (3–5)	4 (4–5)	5 (4–6)	5 (4–6)	5 (5–6)
B-Blood vessels								
1-Thrombi	1 (1–2)	2 (1–3)	3 (2–3)	2 (1–4)	3 (2–3)	3 (2–4)	3 (2–4)	2 (2–3)
2-Parasites	1 (1–2)	1 (0–4)	2 (0–2)	1 (0–2)	1 (0–2)	2 (1–2)	1 (1–2)	1 (1–2)
C-Renal tubules								
1-Tubulonephrosis & necrosis	3 (2–4)	3 (3–4)	3 (2–4)	2 (1–2)	2 (2–3)	3 (2–3)	0 (0–1)	0 (0–1)
2-Nephron neogenesis	1 (1–2)	1 (0–1)	2 (1–3)	3 (2–4)	3 (2–4)	4 (4–5)	5 (5–6)	5 (5–6)
3-Intraluminal stages of parasite	0 (0–0)	0 (0–0)	0 (0–0)	0 (0–0)	0 (0–0)	0 (0–0)	0 (0–0)	0 (0–0)

Numbers in parentheses indicate the lowest and highest values counted in 10 microscopic fields (400×), based on the following scoring criteria: 0, no lesions; 1, minimal lesions; 2, mild lesions; 3, mild to moderate lesions; 4, moderate lesions; 5, moderate to severe lesions; 6, severe lesions. Different letters a, b in the same raw for each time point separately were significant at *P* < 0.05

**Fig. 4:**
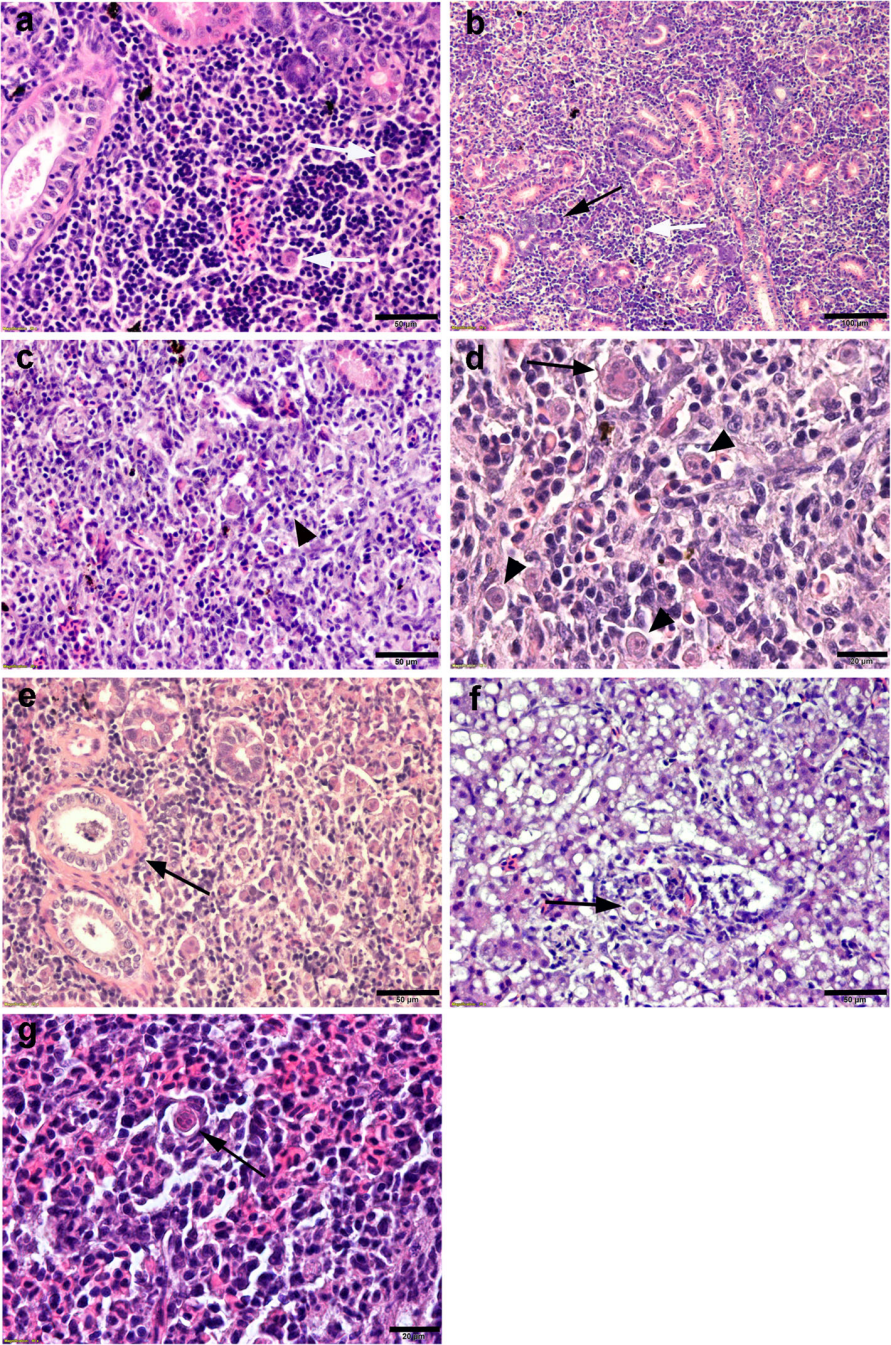
Comparative histopathology of PKD on rainbow trout during single and co-infection with WD. **a** Kidney section of single *Tb* infected group showing interstitial extrasporogonic stages of *T. bryosalmonae* surrounded by macrophage cells (rosette appearance, *arrow*) at 60 dpe. **b** Kidney section of single *Tb* infected group at 120 dpe, showing pronounced tubular regeneration and tubuloneogenesis with basophilic cytoplasm and increased numbers of mitotic figures (*black arrow*), note interstitial *Tb* stages (*white arrow*). **c-e** *Mc*-then *Tb* co-infected fish, 120 dpe to *Mc* (90 dpe to *Tb*), showing **c** expanded interstitium due to advanced fibrosis with infiltration of inflammatory cells (*arrowhead*), **d** higher magnification of (**c**) showing presence of mutinucleated giant cell (*arrow*) and the parasites (*arrowheads*) and **e** peritubular fibrosis (*arrow*). **f** Liver section of single *Tb* infected group at 120 dpe showing presence of inflammatory cellular aggregations containing few parasite stages (*arrow*). **g** Spleen of single *Tb* infected group at 120 dpe showing presence of *T. bryosalmonae* stages (*arrow*) in red and white pulp associated with inflammatory cellular infiltration around the zone of infection. H&E staining. *Scale-bars*: **a**, 50 μm; **b**, 100 μm; **c**, 50 μm; **d**, 20 μm; **e**, 50 μm; **f**, 50 μm; **g**, 20 μm

At 60 dpe, the renal interstitial hematopoietic tissue of single *Tb* infected group showed a mild hyperplasia, associated with moderate infiltration of macrophages and lymphocytes (grade 1–2). Interstitial extrasporogonic stages of *T. bryosalmonae* were surrounded by macrophage cells in a typical rosette appearance (Fig. 4a) and associated with degenerated and necrotic changes of renal tubular epithelia with a presence of hyaline droplets and casts. Some tubules showed the presence of interepithelial parasite stages. No significant differences in the histopathological changes of kidneys of all *Tb* infected groups at 60 dpe. Parasite numbers were higher in *Mc*-then *Tb* co-infected group than single *Tb* infected and *Tb*-then *Mc* co-infected groups.

At 90 and 120 dpe, up to 50% of kidney in single *Tb* infected and *Tb*-then *Mc* co-infected groups showed lesions of moderate nephritis. Moderate infiltration of chronic inflammatory cells with moderate fibrosis resulted grade 2. Many tubular epithelia showed marked hypercellularity with high mitotic figures at 120 dpe (Fig. 4b).

Whereas in *Mc*-then *Tb* co-infected group more than 50% of kidney showed marked nephritis and fibrosis with infiltration of multinucleated giant cells, macrophages, lymphocytes and epithelioid cells (Fig. 4c, d). A marked peritubular fibrosis was also observed (Fig. 4e).

Liver sections from all *T. bryosalmonae* infected groups showed the presence of multiple foci of inflammatory cellular aggregations containing few parasite stages especially in hepatic sinusoids and sometimes in central veins (Fig. 4f). Spleens showed also presence of moderate numbers of parasite stages in red and white pulp associated with inflammatory cellular infiltration around the zone of infection (Fig. 4g). Spores of *T. bryosalmonae* were not detected in brain, heart and gill sections either in single *Tb* infected or co-infected groups at all time points.

**Fig. 5:**
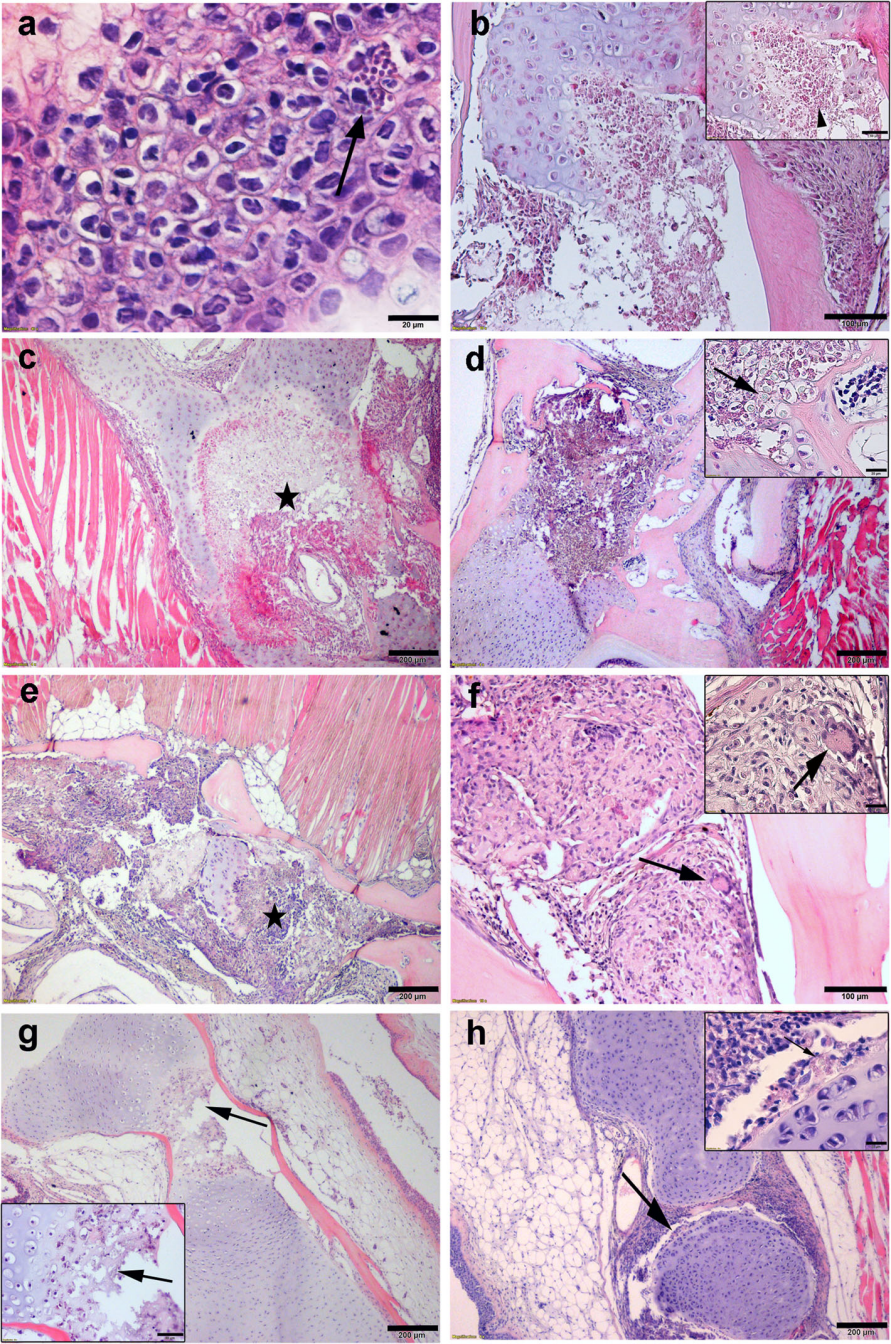
Comparative histopathology of WD on rainbow trout during single and co-infection with PKD. **a** Epidermis showing numerous intracellular aggregates of dark stained sporoplasms between the epithelial cells 30 min post-exposure to *M. cerebralis* (*arrow*). **b** Cranium of single *Mc* infected group, 60 dpe shows presence of cartilaginous necrotic foci containing numerous developmental stages of parasite (inset, *arrowhead*). **c** Cranium of *Mc*-then *Tb* co-infected group, 60 dpe to *Mc* (30 dpe to *Tb*) showing severe cartilage destruction involves most of the cartilage and infiltration of inflammatory tissues (*star*). **d** Single *Mc* infected group at 90 dpe showing presence of numerous mature myxospores in the area of cartilage necrosis (inset, *arrow*) with infiltration of macrophages and lymphocytes. **e** *Mc*-then *Tb* co-infected group, 90 dpe to *Mc* (60 dpe to *Tb*) showing severe destruction of cranial cartilage with displacement (*star*). **f** Affected cranial cartilage of single *Mc* infected group at 120 dpe showing presence of multinucleated giant cells in the site of granulomatous inflammation (*arrow*). **g** Cranium of *Tb*-then *Mc* co-infected group, 90 dpe to *Tb* (60 dpe to *Mc*) showing presence of small discrete foci of degenerated cartilage (*arrow*) and containing few generative stages of *M. cerebralis* with few leukocytes (inset, *arrow*). **h** Cranium of *Tb*-then *Mc* co-infected group, 120 dpe to *Tb* (90 dpe to *Mc*) showing presence of mild cartilagneous degeneration and necrosis with few chronic inflammatory cells surrounding the affected cartilage (*arrow*) and presence of few mature myxospores (inset, *arrow*). H&E staining. *Scale-bars*: **a**, 20 μm; **b**, 100 μm (inset 50 μm); **c**, 200 μm; **d**, 200 μm (inset 20 μm); **e**, 200 μm; **f**, 100 μm (inset 20 μm); **g**, 200 μm (inset 50 μm); **h**, 200 μm (inset 20 μm)

The penetrating stages of *M. cerebralis* were detected in the epidermis at 30 min post-exposure and intracellular aggregates of dark stained sporoplasms were seen between the epithelial cells in the dermal layer (Fig. 5a). First observation of developmental stages of *M. cerebralis* was seen in cranium at 30 dpe. Cartilage tissues at this period showed a small area of necrosis with few developmental stages of *M. cerebralis*.

At 60 dpe, cranial lesions in single *Mc* infected group were grade 3 and characterized by the presence of cartilaginous necrotic foci containing numerous vegetative cells and developmental stages of the parasite (Fig. 5b). The lesions were infiltrated with large numbers of inflammatory cells mainly macrophages and lymphocytes with a beginning of fibrous tissue infiltration. Whereas the cranial lesions were grade 4 in *Mc*-then *Tb* co-infected group with severe cartilage destruction involves most of the cranial cartilage with infiltration of inflammatory cells (Fig. 5c).

At 90 dpe, with the progressing of WD, lesions in single *Mc* infected fish became chronic in nature with the presence of numerous mature myxospores and pansporoblasts stages of *M. cerebralis*, grade 3*.* Infiltration of fibrous tissues and chronic inflammatory cells (macrophages, lymphocytes, plasma cells and fibrocytes) into the affected cartilage were seen (Fig. 5d). The lesions in *Mc*-then *Tb* co-infected group were also chronic and still more severe (grade 4) with massive areas of cartilaginous necrosis and displacement. Infiltration of a large amount of fibrotic tissues and chronic inflammatory cells which replaced the affected part of cartilage was seen (Fig. 5e). Gill arch in all *M. cerebralis* infected groups also showed the presence of mature myxospores associated with inflammatory cell infiltrations and necrosis of cartilaginous tissues.

**Fig. 6:**
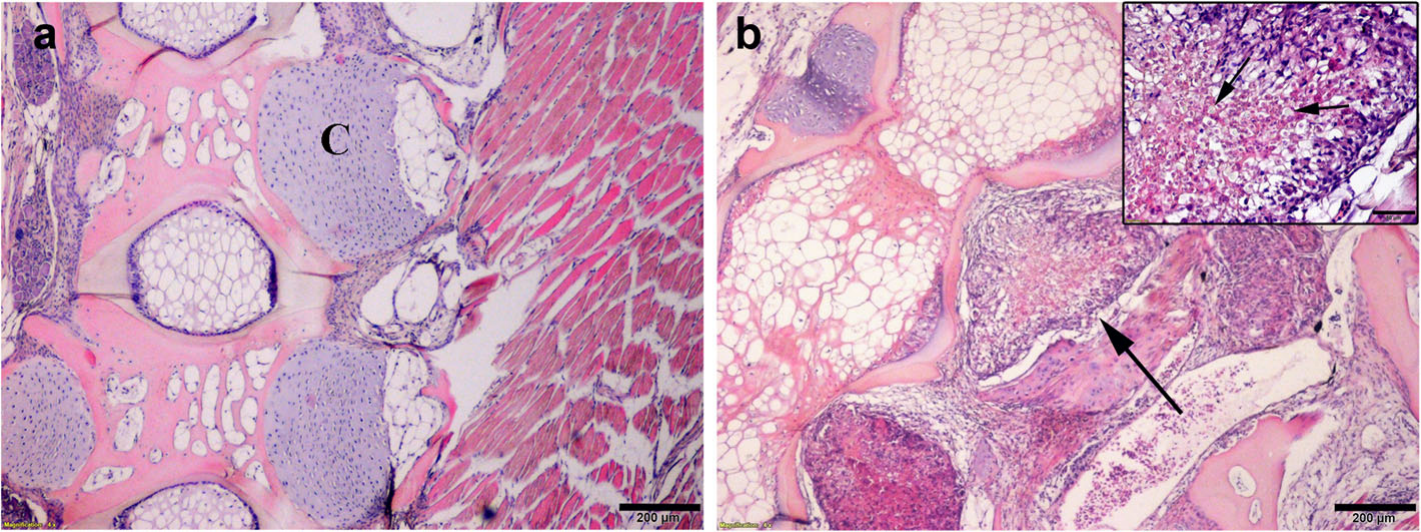
Histopathology of vertebral column during co-infection. **a** Uninfected control fish show normal spine with normal cartilage (C). **b** Spine of *Mc*-then *Tb* co-infected group, 120 dpe to *Mc* (90 dpe to *Tb)* with severe cartilage necrosis, infiltrating inflammatory cells (*arrow*) and presence of mature myxospores (inset, *arrow*). H&E staining. *Scale-bars*: **a**, 200 μm; **b**, 200 μm (inset 50 μm)

At 120 dpe, the most characteristic feature was the appearance of multinucleated giant cells in the site of granulomatous inflammation of the affected cartilage (Fig. 5f). Bony tissues were replaced by the formed granulomas leading to destruction of the structural framework and replacement. Fish showed permanent skeletal disfigurations and caudal curvatures which were more obvious at this stage mostly in fish from *Mc*-then *Tb* co-infected group. Granulomatous reactions were also observed in the cartilages of vertebral column and most of them were destroyed and containing a large number of myxospores (Fig. 6b). On the contrary, the pathological lesions of WD in *Tb*-then *Mc* co-infected group were less and showed minimal infection, grade 1. And small discrete foci of degenerated cartilage containing few numbers of generative stages of *M. cerebralis* with few infiltrated leukocytes were observed at 60 and 90 dpe to secondary *M. cerebralis* (Fig. 5g, h).

### Immunohistochemistry

**Fig. 7:**
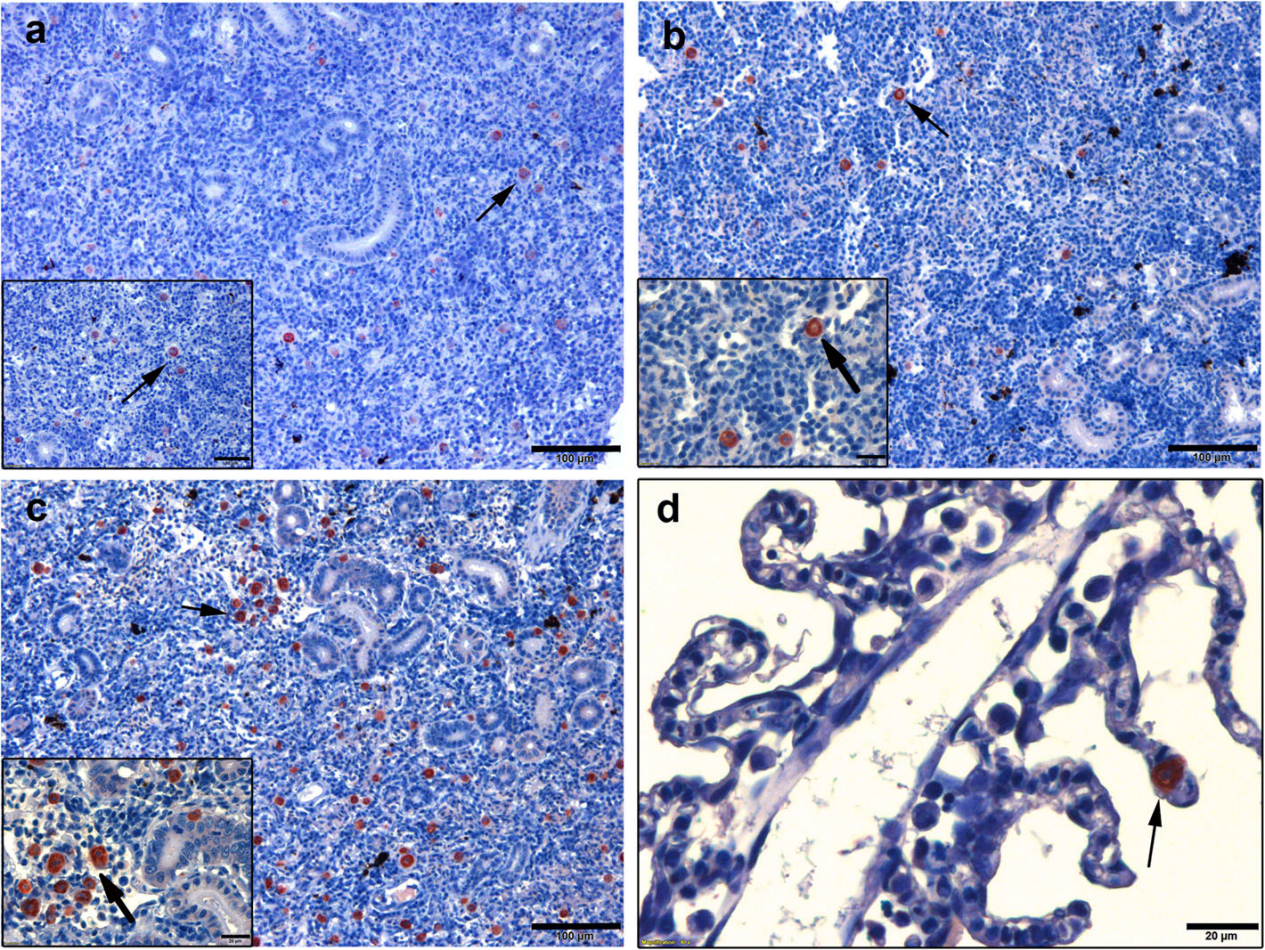
Comparative immunohistochemistry of PKD on rainbow trout kidney and gills during single and co-infection with WD. **a**, **b** Kidney sections of single *Tb* infected and *Tb*-then *Mc* co-infected groups respectively at 90 dpe showing moderate numbers of interstitial extrasporogonic stages of *Tb* (*arrow*). **c**, **d** *Mc*-then *Tb* co-infected group, 120 dp initial exposure to *Mc* (90 dpe to secondary *Tb*), (**c**) kidney section showing large number of interstitial extrasporogonic stages of *Tb* distributed in most of kidney interstitium (*arrow*), (**d**) gill section showing presence of one parasite stage of *T. bryosalmonae* in lamellar epithelium (*arrow*). *Scale-bars*: **a**, 100 μm (inset 50 μm); **b**, 100 μm (inset 20 μm); **c**, 100 μm (inset 20 μm); **d**, 20 μm

**Fig. 8:**
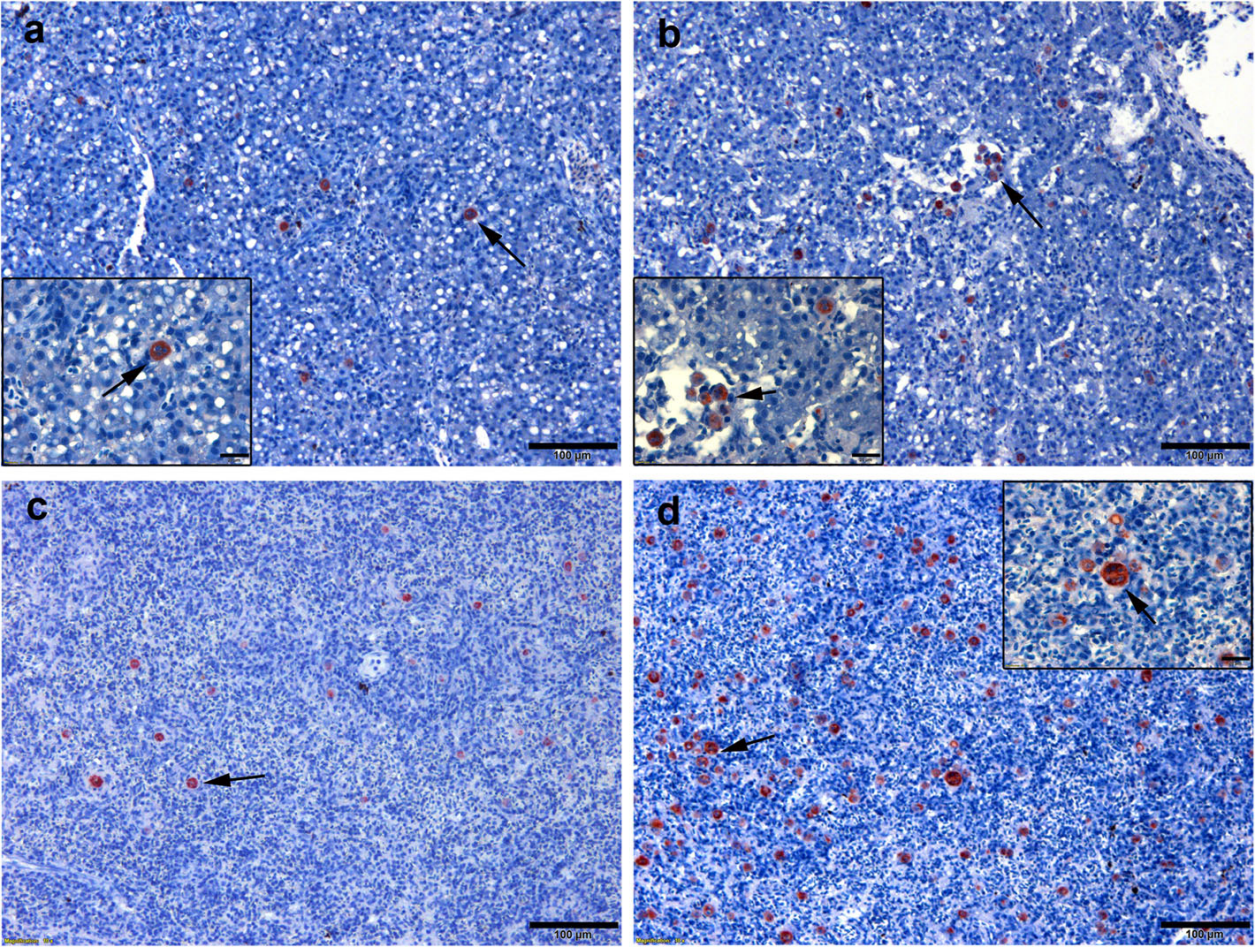
Comparative immunohistochemistry of PKD on rainbow trout liver and spleen during single and co-infection with WD. **a** Liver section of single *Tb* infected group, 90 dpe showing presence of few parasite stages in hepatic parenchyma (*arrow*). **b** Liver section of *Mc*-then *Tb* co-infected group, 120 dp initial exposure to *Mc* (90 dpe to secondary *Tb*) showing presence of many parasite stages in hepatic parenchyma and central veins (*arrow*). **c** Spleen section of single *Tb* infected group, 90 dpe showing presence of few parasite stages (*arrow*). **d** Spleen section of *Mc*-then *Tb* co-infected group, 120 dp initial exposure to *Mc* (90 dpe to secondary *Tb*) shows presence of many parasite stages in the parenchyma (*arrow*) associated with horseshoe-shaped multinucleated giant cell, strong positively IHC stained (inset, *arrow*). *Scale-bars*: **a**, 100 μm (inset 20 μm); **b**, 100 μm (inset 20 μm); **c**, 100 μm; **d**, 100 μm (inset 20 μm)

Table 1 shows numbers of *T. bryosalmonae* in different kidney compartments of single *T. bryosalmonae* infected and other co-infected groups. In kidneys, higher numbers of the parasite were observed in the interstitial space and in blood vessels of fish from *Mc*-then *Tb* co-infected group when compared to those from single *Tb* infected or *Tb-*then *Mc* co-infected groups at 60 and 90 dpe (Fig. 7a-c). Parasite stages were also detected in the gill epithelia of *Mc*-then *Tb* co-infected fish at 90 dpe to secondary *T. bryosalmonae* (Fig. 7d). In liver, at 90 dpe *T. bryosalmonae* spores were mainly observed in the central veins and hepatic sinusoids in association with macrophages and lymphocytes. The numbers of the parasite were higher in *Mc*-then *Tb* co-infected group than single *Tb* infected and *Tb-*then *Mc* co-infected groups (Fig. 8a, b). Examination of spleen sections at 90 dpe revealed presence of developmental stages of the parasite in the interstitial hematopoietic tissues which were in higher numbers in *Mc*-then *Tb* co-infected group than other groups (Fig. 8c, d). Moreover, horseshoe-shaped multinucleated giant cells were seen in the spleens from *Mc*-then *Tb* co-infected group showed a strong positive signal (Fig. 8d). No parasite stages were detected at any time points in heart or brain sections.

## Discussion

Animal diseases studies often focus on single infections with selected host-pathogen models; however, co-infections are common to occur in nature [25, 26, 43]. Co-infection studies are still in their beginning, although limited established models increased demands towards more research in this direction. Multi-parasitic interactions have a strong effect on infection dynamics and play a role in shaping parasite and host populations [43]. The present study describes for the first time a co-infection model with two different myxozoans in rainbow trout, providing novel observations on their impact on mortality rates and pathological lesions. During the experimental period all parameters (water flow rate, temperature and photoperiod applied) were the same for all tanks. Although there were not replicate tanks for the difference exposure regime, we therefore conclude that differences in the mortality rate, body lengths and weights or histopathological lesions were due to the effect of exposure to the parasites in the single and co-infected groups, and not tank effects.

Physical parameters (weights and lengths) were significantly affected at 90 and 120 dpe in *Mc*-then *Tb* co-infected group compared to single *Tb*, single *Mc* and *Tb*-then *Mc* co-infected groups. The mortalities were significantly higher in *Mc*-then *Tb* co-infected group when compared to other infected groups. The mortality rates are in accordance with previous records, including that higher mortality occurred during co-infection either with homologous or heterologous or opportunistic secondary pathogens [44, 45]. Gorgoglione et al. [8] also found that highest mortalities (100%) were reached when PKD-infected juvenile brown trout were naturally concurrently infected with common ectoparasites, including the ciliated protozoans *Ichthyophthirius multifiliis*, *Chilodonella uncinata* and the monogeneans *Gyrodactylus derjavini*.

We found an exacerbated pathogenesis eliciting more serious clinical signs and pathological lesions of PKD and WD in *Mc*-then *Tb* co-infected group, when compared to the single infections with *T. bryosalmonae* or *M. cerebralis.* This co-infected group showed enlarged kidneys and spleens at 60–120 dpe with a higher kidney swelling index (grade 4), and lesions scores and *T. bryosalmonae* numbers were significantly higher in the kidneys, spleens and livers as seen by immunohistochemistry. Additionally, this co-infected group also exhibited severe lesions of WD (grade 4) with more severe cartilage destruction and displacement and showed severe skeletal deformities and caudal curvature compared to single *M*c infected group that showed the lesions but in a lower extent, grade 3. It suggests that severe form of both diseases in co-infected fish may be due to immunosuppression by *T. bryosalmonae*. PKD is a strong immunosuppressive disease for rainbow trout, thus it increases the susceptibility to opportunistic pathogens upon natural outbreaks [8, 21]. These results are also in agreement with Schmidt-Posthaus et al. [14] describing that the recovery from PKD in brown trout was depending on the presence or absence of concurrent infection with the larvae of the nematode parasite, *Raphidascaris acus*. Furthermore, the regeneration of the kidney parenchyma was not complete with moderate tubulonephrosis, and chronic renal lesions in the presence of the parasite, whereas, the kidney recovered completely in the absence of the parasite. During the primary infection of rainbow trout with *M. cerebralis*, triactinomyxon–sporoplasms penetrate the epidermis and dermis, then through the peripheral and central nervous system to reach the cartilage during the first 30 days [24, 46]. Rationally, it seems that the triactinomyxon spores utilize this penetrating way just to escape from the immune response and to more efficiently multiply to higher numbers before reaching the cartilage as the target site. This is considered one of the reasons that make WD difficult to control [46]. During PKD some key regulatory cytokines are downregulated with a significant decrease in the phagocytic activity of granulocytes, leading to the immunopathological condition [21, 47]. Holland et al. [48] also reported that PKD-infected fish become stressed and exhibit increase susceptibility to secondary infections through stress/cortisol-mediated immune cytokine suppression. Therefore, our explanations of the synergistic interaction occurred in *Mc*-then *Tb* co-infected group, may be due to evasion of the developmental stages of *M. cerebralis* from the immune system and from the strong immunosuppression effect induced by secondary *T. bryosalmonae*. Therefore, fish exhibited the lesions of WD together with the lesions of PKD in an exaggerated form.

Conversely, *Tb*-then *Mc* co-infected group exhibited typical pathological changes of both parasitic diseases, but with a lower mortality rate similar as caused by the single *T. bryosalmonae* or *M. cerebralis* infection. The clinical signs and lesions of WD were milder with only slight whirling movements and without skeletal deformities. While kidney swelling index of co-infected fish was similar to single *T. bryosalmonae* infection. We found during this co-infection that secondary infection by *M. cerebralis* resulted in antagonistic interaction and decreased in the fish susceptibility to whirling disease. Anti-*T. bryosalmonae* antibodies were detected in fish as early as 6 weeks post-exposure [6, 49]. PKD pathogenesis is characterized by a profound B/antibody response with upregulation of secretory form of IgM and IgT, which positively correlated with *T. bryosalmonae* prevalence [9, 21]. Anti-*T. bryosalmonae* monoclonal antibody B4 was found to cross react with sporogonic stage of *M. cerebralis* [50]. The presence of cross-reaction between both parasites could induce a cross-immunity which counteracts with the pathogenesis of secondary WD, suggesting the presence of common antigen in myxozoans [51, 52]. Hedrick et al. [46] referred to the importance of the first 2–4 dpe to *M. cerebralis* in the development of WD and this support our explanations. Based on the previous studies, we suggest that primary infection with *T. bryosalmonae* may have a role to trigger the innate and adaptive immune response to subsequent infection with *M. cerebralis*. This antagonistic interaction in such case is similar to the negative association between *T. bryosalmonae* and *C. schurovi* during mixed infection where infection by one parasite could lower the probability of infection by the second [29, 30]. Therefore, antagonistic interactions could occur during multi-parasitism as in the results of Brutus et al. [53] and Naus et al. [54] where they found a clear negative association during co-infections with *Ascaris* and malaria parasite or *Schistosoma* and *Plasmodium*, respectively.

No stages of *T. bryosalmonae* were detected in H&E stained gill sections of all groups at all time points. However, the immunhistochemical staining showed the presence of some parasite stages in the gill lamellar epithelia of *Mc*-then *Tb* co-infected fish. Brain and heart sections from all groups did not exhibit any presence of spores neither in H&E or IHC stained sections. These results were similar to the results mentioned by Abd-Elfattah et al. [55] who did not find any *T. bryosalmonae* in brain or intestine sections of chronically infected brown trout. The presence of horseshoe-shaped multinucleated giant cells with strong positive IHC reaction in the spleen at 90 dpe of *Mc*-then *Tb* co-infected fish indicated their phagocytic immune role and the immune defence role of the spleen in case of PKD infection to eliminate the parasite. This was in accordance with Morris et al. [56] who found occasionally phagocytes containing degenerated secondary cells of *T. bryosalmonae* via using mAb B4.

## Conclusions

To our knowledge, our study presents for the first time from laboratory infections, the impact of co-infections with myxozoans, *T. bryosalmonae* and *M. cerebralis*, on rainbow trout. The primary *Mc* infection followed by *Tb* had the most serious impact on fish, because of the induced synergistic interaction. The contrary instead occurred in *Tb*-then *Mc* co-infection, as signs of WD were diminished and mortality was not affected. Fish showed lesions of PKD as single *T. bryosalmonae*-infected group. More research would be required to assess the effects of myxozoan infection interactions on the quality of the immunological response differentially elicited in co-infected rainbow trout as a tool toward the development of efficient disease control strategies.

## Abbreviations

Dpe: Days post-exposure
*Mc*: *Myxobolus cerebralis*
PKD: Proliferative kidney disease
*Tb*: *Tetracapsuloides bryosalmonae*
WD: Whirling disease
